# Counseling on Access to Lethal Means-Emergency Department (CALM-ED): A Quality Improvement Program for Firearm Injury Prevention

**DOI:** 10.5811/westjem.2020.5.46952

**Published:** 2020-08-20

**Authors:** Kristen L. Mueller, Sonya Naganathan, Richard T. Griffey

**Affiliations:** * Washington University in St. Louis School of Medicine, Department of Emergency Medicine, St. Louis, Missouri; † Warren Alpert Medical School of Brown University, Department of Emergency Medicine, Providence, Rhode Island

## Abstract

**Introduction:**

Suicide is the 10^th^ leading cause of death in the United States, with firearms reported as the cause of death in up to 50% of these cases. Our goal was to evaluate the feasibility of the Counseling on Access to Lethal Means intervention in the Emergency Department (CALM-ED) by non-physician personnel.

**Methods:**

We conducted this single-center, prospective, quality improvement study (QI) in an urban, academic ED with over 90,000 annual patient visits. The study looked at adult patients who were discharged after presenting to the ED with suicidal crisis. Assessment of access to lethal means was conducted at the bedside, followed by a counseling session regarding safe storage of lethal means and follow-up via telephone call 48–72 hours after ED discharge. We collected data on patient’s sociodemographics, psychiatric history, access to lethal means, lethal means storage methods, the patient’s specific plans for lethal means storage after discharge, and post-discharge follow-up care.

**Results:**

Of 215 eligible patients, 166 voluntarily agreed to participate in CALM-ED, of whom 84 (51%) reported access to lethal means. Following the intervention, 75% of patients described a specific storage plan for their lethal means. Patients with and without access to firearms were equally likely to participate in the follow-up telephone call.

**Conclusion:**

An ED-based CALM QI intervention is feasible for implementation by non-physician personnel and is well received by patients and families. This intervention has the potential to help saves lives at times of suicide crisis.

## INTRODUCTION

Firearm-related injury and death represent a public health epidemic. Suicide is the 10^th^ leading cause of death in the United States, with firearms reported as the cause of death in up to 50% of these cases.^1^ Up to 90% of suicides attempted with a firearm result in death, and the direct medical costs resulting from firearm injures are as high as $2.9 billion dollars per year.^2^,^3^ Additionally, many patients have their first point of contact with the mental healthcare system less than one month before suicide is attempted.^4^

While these findings suggest that many patients would benefit from outpatient mental healthcare, over 100 million people in the US live in a mental health-designated Health Professional Shortage Area, in which only 27% of the mental healthcare need is met.^5^ Consequently, the emergency department (ED) is a frequent point of access to care for patients with mental health crises, with nearly 1% of all US ED visits in 2013 involved in evaluation and management of suicidal ideation (SI).^6^ In bridging the gap to accessible mental healthcare services, there is an opportunity to improve safety from suicide in ED patients through counseling on safe storage of lethal means at times of suicidal crisis.

The majority of the health professional associations that deal directly with mental healthcare have endorsed suicide risk assessment and counseling on access to lethal means, such as safe firearm storage practices.^7^,^8^ Multiple studies have shown that counseling on safe storage of lethal means can improve safe firearm storage, and thus decrease risk of firearm-related suicide.^9^–^14^ Despite this finding, routine lethal-means counseling for at-risk patients has not been widely adopted in high-volume settings such as the ED. In 2013, Betz et al found that only 22% of emergency providers regularly assess for firearm access in patients with SI.^15^ And a two-year survey of ED nurse managers at facilities that discharged suicidal patients identified significant gaps in asking about firearm access and counseling on safe storage when patients reported access.^16^ A similar needs-assessment study conducted at our institution found that emergency physicians documented access to firearms in only 3% of suicidal patient encounters.^17^ This gap inspired a project to counsel patients on access to, and safe storage of, lethal means—especially at times of suicidal crisis.

In 2014 we launched a university-wide gun violence strategic initiative to identify gaps in available research and find actionable measures to reduce gun violence.^18^ One result of this effort was the development of the Counseling on Access to Lethal Means (CALM) quality improvement (QI) intervention, “CALM-ED,” based upon the free, online CALM module available through the Suicide Prevention Resource Center.^19^ This online module was designed specifically for mental health and medical providers who counsel people at risk for suicide. A growing body of literature, including a recent survey of community-based mental healthcare workers’ knowledge and attitudes after completing an in-person CALM workshop, demonstrated a positive association between CALM training and comfort discussing CALM, and suggests that participating in CALM training improves provider self-reported comfort and increased frequency in use of CALM.^20^ However, there is a paucity of evidence on how to systematically provide this intervention across clinical settings.^21^,^22^ Indeed, in 2018 Betz et al reported that among 800 ED patients who screened positive for suicide risk, only 18% had a documented assessment of access to lethal means, and only 8% had documentation of a specific plan to reduce access to said lethal means.^23^

Leveraging the accessibility of CALM and the abundance of at-risk patients in our ED population, while cognizant of the time demands on emergency physicians, we developed the QI program CALM-ED, which was implemented by non-physician personnel. This work serves as a primer on how to improve frequency of real-time lethal means counseling among the highest risk suicidal patients in the ED setting. As a first step, in this study we evaluated the feasibility of non-physician providers successfully delivering this intervention in the ED. We also aimed to better stratify the at-risk population of suicidal patients presenting to the ED and evaluate whether participating in CALM-ED leads to increased compliance with outpatient mental healthcare and safe storage of lethal means.

**Population Health Research Capsule**

What do we already know about this issue?

*Suicide is the 10^th^ leading cause of death in the US, with firearms the reported cause of death in up to 50% of these cases.*

What was the research question?

Can the suicide prevention strategy Counseling on Access to Lethal Means be implemented in the emergency department (CALM-ED)?

What was the major finding of the study?

Following the CALM-ED intervention, the majority of patients described a specific storage plan for their lethal means.

How does this improve population health?

An ED-based CALM intervention is feasible and well received by patients and families. CALM has the potential to help save lives at times of suicide crisis.

## METHODS

### Study Design

This was a single-center, prospective QI study evaluating the feasibility of delivering counseling to patients presenting to the ED with suicidal ideation. Findings are reported in accordance with SQUIRE (Standards for Quality Improvement Reporting Excellence guidelines).^24^,^25^ This study was approved by our institutional review board (IRB) and included a waiver of consent.

### Study Setting and Population

We conducted this study between January 1, 2018–June 5, 2019, in an urban, academic ED with over 90,000 annual patient visits. Patients were eligible for inclusion if they met the following criteria: English-speaking; 18 years or older; had nursing-assigned triage chief complaints of “suicidal ideation,” “suicidal attempt,” or “depression;” had been placed on suicidal elopement precautions; had access to a telephone; and were ultimately discharged from the ED. We excluded patients who were admitted to the hospital, actively psychotic, or refused the intervention. Patients who arrived intoxicated were eligible once clinically sober. Patients received usual care of psychiatric conditions in the ED, which was provided by attending and resident emergency physicians. Some patients received consultation by the psychiatry service at the discretion of the ED team in the course of their usual care; psychiatric consultation was not part of the CALM-ED intervention. If psychiatry was consulted, patients only received CALM-ED if they had subsequently been cleared for discharge. Both the ED team and psychiatry consultants could provide CALM as part of their standard care. Eligible patients received CALM-ED from the study team regardless of other ED care provided.

### Intervention Counselors

Research coordinators from our Emergency Care Research Core (seven registered nurses, one respiratory therapist, and two college-educated coordinators) completed training on administration of the CALM-ED intervention through the online CALM module available through the Suicide Prevention Resource Center, use of scripted language, and direct teaching by the study team.^19^ These intervention counselors were available 24 hours a day/7 days a week for enrollment and delivery of CALM-ED. Use of non-physician counselors was directed at offloading counseling from clinician staff and at making our results more generalizable to non-academic, non-tertiary care settings where nurses, advanced practice providers, medical assistants, behavioral health coordinators, and other non-physician providers may conduct suicide risk assessment. To avoid conflicts of interest, the authors did not hire or manage the coordinators, nor did we directly provide the intervention or data collection for this study. After completing the CALM training, the majority of intervention counselors expressed comfort with their ability to suicidal ideation and CALM with ED patients.^26^

### Identification of Participants

Patients presenting to the ED with SI were identified by CASE-ED (Computer Assisted Screening and Enrollment in the ED). CASE-ED is an IRB-approved, case-finding program that notified intervention counselors via secure text messaging of patients with the aforementioned nursing-assigned, triage chief complaints. The intervention counselors approached the emergency physician team after “usual care” ED evaluation was complete to determine which patients would be discharged home. To protect patient privacy, all interventions occurred in the patient’s treatment room in the ED.

### Intervention Delivery

The intervention counselors performed a short assessment of access to lethal means at the bedside and delivered a brief, scripted counseling session with patients and any family members or friends present regarding safe storage of firearms and other lethal means. This script, which includes stating your care role as part of the ED team, asking the patient directly about access to lethal means and how they are currently stored, and creating an individualized safe storage plan for any lethal means present, is provided in Appendix Figure 1. As CALM is supported by national organizations and practice guidelines and a valuable care service that has previously been difficult to operationalize in the ED setting, the intervention counselors identified themselves as part of the care team.^27^–^29^ Participants were given handouts developed in partnership with our Institute for Public Health (Appendix Figure 2) detailing mental health resources and local options for safe storage of firearms, and patients who possessed firearms were also given free gun locks upon discharge.

A scripted follow-up via telephone call occurred 48–72 hours after ED discharge. Telephone calls were made to the phone numbers provided to the intervention counselors by patients at the time of discharge. A standardized, follow-up telephone call script was followed. Patients who responded that they were not following the safe practices discussed while in the ED were reminded of the importance of doing these things for their safety. Additionally, if a patient endorsed active suicidality at the time of the follow-up call, a warm handoff was conducted between our intervention counselors and the crisis hotline counselors at a local mental healthcare organization.

### Data Collection

We collected data including patient age, gender, race, marital status, substance use, psychiatric history, personal history of suicide attempt, and whether this ED visit was for a suicide attempt. Additionally, we collected data on the following: access to lethal means (firearms, pills, other); how lethal means were stored; patient’s specific plans to store lethal means after discharge; and patient and/or family/friend phone contact information for follow-up phone calls. All intervention counselors were trained in data collection and were supervised in their baseline performance of the intervention by the lead intervention counselor for this study. We collected and managed study data using REDCap version 7 (Vanderbilt University, Nashville, TN) electronic data capture tools.

Intervention counselors made up to three follow-up calls, inquiring whether and how lethal means were stored safely, whether the patient had established outpatient follow-up care (primary doctor, psychiatrist, therapist, or other), assessed for barriers to safe storage of lethal means, and inquired whether the patient was actively suicidal.

### Outcome Measures

The primary outcome of this study was feasibility of implementation of the CALM-ED intervention in the ED by non-physician providers. This was informed by prior description of feasibility as an implementation outcome and included 1) the success of counselors in completing enrollment and all elements of CALM-ED implementation for the majority of eligible patients; and 2) enrolled patients’ ability to state a plan for safe storage of lethal means (when applicable) prior to discharge.^30^ Secondary outcomes – including better identification of patient demographics in this cohort; past psychiatric history including suicide attempt; substance use history; and types of lethal means patients had access to and their current storage practices, and current outpatient mental healthcare resources – were assessed through completion of a standardized data collection tool.

### Analysis

We present descriptive statistics for participating sociodemographic data and self-reported outcomes. Categorical data are presented as counts and proportions. Chi-square analysis was used to evaluate the difference in follow-up participation for firearm owners vs non-firearm owners. We analyzed data using IBM SPSS Statistics for Windows, version 26 (International Business Machines Corporation, Armonk, NY).

## RESULTS

**Figure 1:**
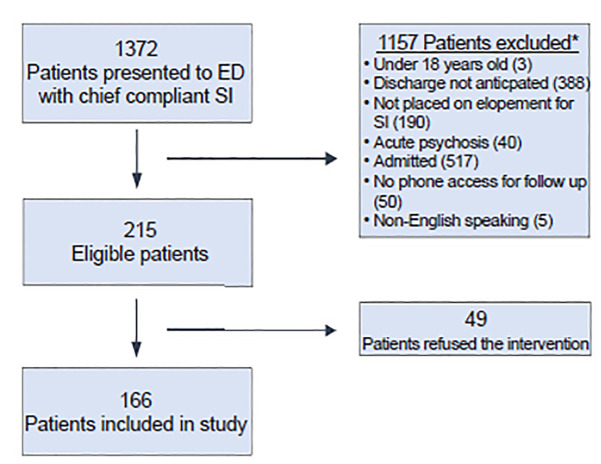
Cohort inclusion matrix of 1,372 patients who presented to the emergency department with SI between January 1, 2018 and June 5, 2019. *some patients met more than 1 criteria for exclusion. *ED*, emergency departmnet; *SI*, suicidal ideation.

We approached all 215 patients meeting eligibility criteria, of whom 166 (77%) voluntarily agreed to participate in CALM-ED (Figure 1). Patient demographics are described in Table 1.

**Figure 2:**
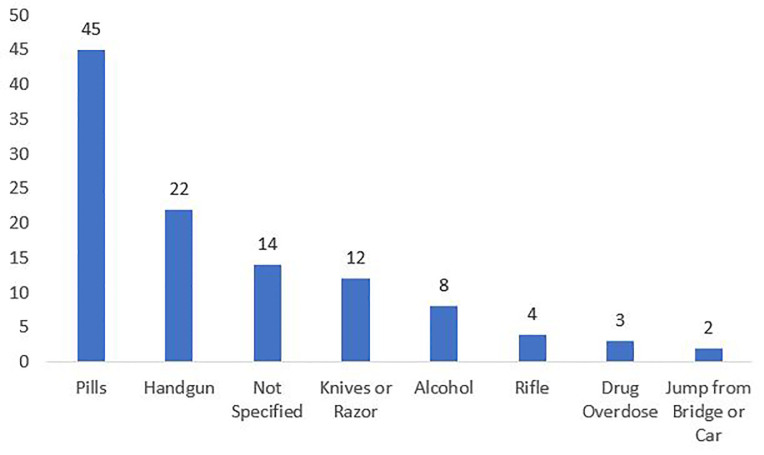
Reported lethal mean access by type.* *some patients reported access to more than one lethal mean.

Of the 166 patients who received CALM-ED, 84 (51%; 95% confidence interval [CI], 0.43 – 0.58) reported access to lethal means. The other 82 patients denied access to lethal means. These included handguns, rifles, alcohol, medications, and “other” – primarily knives, jumping off a bridge or out of a car, and street drug overdose (Figure 2). Twenty-three (13.9%) patients in this cohort reported access to firearms; their pre-intervention storage methods are reported in Table 2. After receiving CALM-ED, of the 84 patients who reported having access to lethal means, 63 (75%; 95% CI 0.64 – 0.83) patients described a specific storage plan for their lethal means after discharge; one reported not having a safe storage plan, and data were missing for 20 eligible patients (Table 3). Eighty-two patients denied access to any lethal means during the CALM-ED intervention. Two patients had their disposition changed after CALM-ED: one patient received a psychiatric consultation, and another received continued ED observation. Psychiatry was consulted for 37 patients. Free gun locks were distributed to 45 of these patients during the intervention period.

Table 3 also details reported follow-up outcomes: follow-up phone calls were completed for 51 patients (31%; 95% CI, 0.24 – 0.38). Patients with and without access to firearms were equally likely to participate in the follow-up telephone call (Pearson chi-square 2.230, p 0.135). Of these 51 patients, 17 (33%; 95% CI, 0.22 – 0.47) reported safely storing their lethal means; 24 (47%; 95% CI, 0.34 – 0.60) denied safe storage practices; and 10 (20%; 95% CI, 0.10 – 0.33) denied access to lethal means before or after CALM-ED. Of the 17 patients who safely stored their lethal means, the majority did so by securing them with friends or family members.

Three patients reported continued thoughts of suicide and were provided with additional resources for their mental healthcare; two of these patients were connected with the crisis line, and the third reported he was comfortable waiting until his upcoming scheduled appointment with his psychiatrist. Twenty-five patients (49%; 95% CI, 0.36 – 0.62) had arranged outpatient, follow-up care with a primary doctor, psychiatrist or therapist at time of follow-up telephone call.

Common barriers to safe storage of lethal means at follow-up included, “my lethal means are household items like knives;” and “I have no one to store my medications.” Several patients expressed appreciation for our concern about their safety, as well as the reminders to store their lethal means safely and schedule outpatient follow-up appointments.

## LIMITATIONS

This was a single-center study in an academic ED, with the limitations associated with this design. The majority of patients who received this intervention were aged 27–51, single, male, Black or White, with a history of substance use and pre-existing psychiatric diagnoses. Patient inclusion was limited to English speakers only. More than half of the patients included in this study had a personal history of suicide attempt, and just over half reported access to lethal means, with 23 patients reporting access to firearms. In this pilot feasibility study, patient inclusion was limited to those presenting with a triage complaint of suicidal ideation; it is possible that relevant patients were excluded by this selection criteria. This high-acuity psychiatric population is indicative of our academic, urban ED environment. However, as this intervention is centered on interpersonal conversations and the development of multidisciplinary networks with outpatient mental healthcare systems, there is nothing that precludes generalization of these protocols to inpatient wards, clinic-based practices, and other clinical settings.

The low follow-up telephone call completion rate of 33% highlights an inherent difficulty in tracking the ED population, particularly those with mental health crises, following discharge. These rates are similar to follow up-rates reported for Black (40.0%) and non-Hispanic White (50.9%) ED patients, and on par with the recently reported 30% seven-day follow-up rate in psychiatric patients after discharge from inpatient hospitalization.^31^,^32^ This potential for difficult follow-up, coupled with the murky legal waters of consenting patients to enroll in a study while actively suicidal, were primary considerations in our decision to evaluate the feasibility of CALM-ED prior to attempting a randomized control trial into whether CALM leads to improved safe storage in suicidal patients.

## DISCUSSION

Over an 18-month period non-physician providers successfully delivered CALM-ED to 166 of 215 eligible patients, demonstrating feasibility. It is our hope that the protocols, script, and data presented here will serve to assist others in implementing CALM-ED style interventions in EDs and other high-acuity clinical settings, and build on prior work in the pediatric emergency setting.^13^ Use of non-physician counselors avoids additional burden on physicians and increases generalizability, especially in clinical settings that use non-physician providers to conduct suicide-risk assessment.

This study takes the next step toward filling knowledge gaps previously identified to increase research and integrate patient-centered programming of firearm injury-prevention strategies such as CALM in bedside clinical practice. Encouragingly, after CALM-ED, most participants were able to state a safe storage plan for their lethal means prior to discharge from the ED, and patients who participated in the follow-up telephone phone call were receptive to continued discussion of safe storage practices. Additionally, patients who reported access to firearms were equally likely to participate in a follow-up telephone call as those with access to other types of lethal means. These results suggest that patients with access to firearms are willing to engage in conversations about safe storage of these lethal means at times of suicidal crisis.

Our data do not contain a documented rationale for the lack of lethal means storage plans prior to discharge from the ED in 20 patients who reported having access to said items; it is possible this was limited by intervention counselor comfort with CALM-ED. Survey data of our intervention counselors indicate that while the majority felt comfortable with the CALM-ED intervention, two felt “somewhat uncomfortable” talking to an individual about safe storage of lethal means, and one felt “somewhat uncomfortable” with their ability to effectively counsel patients on reducing access to medications and firearms.^26^ Additionally, one respondent felt “very uncomfortable” with all counseling topics related to suicidal ideation, access to lethal means, and safe storage of lethal means. Alternatively, this discussion regarding patient plans for safe storage may have taken place prior to discharge, but was not documented due to time constraints, incomplete task-switching, or failure of task completion.

While most of our patients with access to lethal means were able to make plans for safe storage, many faced barriers to enacting these plans. As was demonstrated in our data, many patients rely on a social network of friends or families to help store or manage their lethal means. This may be problematic for patients who are socially isolated or live away from their families. Creation of third-party networks for temporary storage of firearms during times of suicidal crisis at police stations, gun ranges, or other repositories could help reduce access to firearms in this high-risk population. Free distribution of gun locks from clinical settings may also help mitigate risk.

Despite our early successes, the suicidal crisis patient population included in this study is just the tip of the iceberg of patients at risk for death by suicide from firearms and other lethal means. As multiple prior studies of discharged ED patients with mental health presentations have documented low follow-up rates, feasibility is a reasonable first step toward a more robust assessment of efficacy. Additional next steps in this work will include expanding CALM-ED to patients with any history of depression or mental health illness (not just acute mental health crisis), substance abuse, and other at-risk populations. Given the many demands on physicians it may be more cost-effective to use non-physician counselors, as proposed in this study, to maximize CALM delivery. Given the ease of administration, especially when provided with scripting, this suggests that non-physician providers can feasibly deliver CALM.

## CONCLUSION

While further study is needed regarding the efficacy of safe storage practices after CALM, especially in the ED population, our findings suggest that an ED-based CALM QI intervention is feasible, well received by patients and families, and has the potential to help saves lives at times of suicide crisis.

## Supplementary Information





## Figures and Tables

**Table 1:** Characteristics of patients with suicidal ideation who received intervention regarding access to lethal means.

Patient characteristics	n (95% CI)
Age in years (mean, IQR)	38 (27–51)
Gender	
Male	102 (0.54–0.69)
Female	64 (0.31–0.46)
Race	
Black	95 (0.50–0.65)
White	64 (0.31–0.46)
Hispanic/Latino	2 (<0.01–0.06)
Other	3 (<0.01–0.05)
Not documented	2 (<0.01–0.06)
Marital status	
Singe	115 (0.62–0.76)
Romantic partner	8 (0.02–0.09)
Married	19 (0.07–0.17)
Divorced	13 (0.05–0.13)
Widowed	3 (<0.01–0.05)
Other	3 (<0.01–0.05)
Not documented	5 (0.01–0.07)
Substance use*	
Alcohol	69 (0.34–0.49)
Cocaine	29 (0.12–0.24)
Marijuana	35 (0.16–0.28)
PCP	1 (<0.01–0.06)
Heroin	19 (0.07–0.17)
Amphetamine	12 (0.04–0.12)
None	43 (0.20–0.36)
Psychiatric history**	
Bipolar	39 (0.18–0.30)
Schizophrenia	37 (0.17–0.29)
Personality Disorder	15 (0.05–0.14)
Depression	69 (0.34–0.49)
Anxiety	41 (0.19–0.32)
None	6 (0.02–0.08)
Not documented	9 (0.03–0.10)
Report established outpatient psychiatric care	71 (0.35–0.50)
History of suicide attempt	110 (0.59–0.73)
Current ED visit for suicide attempt	102 (0.54–0.71)

* Some patients reported use of more than one substance.

** Some patients reported use of more than one psychiatric diagnosis.

*CALM-ED*, Counseling on Access to Lethal Means intervention in the emergency department; *CI*, confidence interval; *IQR*, interquartile ratio; *PCP*, phencyclidine.

**Table 2:** Firearm storage methods.

Storage type	n (%)
Locked in safe	6 (26)
Unlocked and unloaded	1 (4)
Unlocked and loaded	1 (4)
Stored with friends/family	8 (35)
Refused to say	2 (9)
Not sure	1 (4)
Other unspecified storage	1 (4)
Not documented	3 (14)

**Table 3:** Storage plan after CALM-ED and at time of follow-up telephone call.

	After CALM-ED*	On Follow-Up
Storage type	n = 166 (%)	n = 51 (%)
Locked in safe	9 (6)	2 (4)
Use gun lock	2 (1)	0 (0)
Lock in closet	1 (1)	1 (2)
Stored with friends/family	66 (40)	8 (16)
Dispose of pills safely	2 (1)	0 (0)
Other removal from home	4 (2)	1 (2)
Not documented	20 (12)	5 (10)
Denied access to lethal means	82 (49)	10 (20)
Did not store lethal means safely	n/a	24 (47)

* Some reported storage plans for more than one type of lethal means.

*CALM-ED*, Counseling on Access to Lethal Means intervention in the emergency department.
