# Supplementary material for: Counseling on Access to Lethal Means-Emergency Department (CALM-ED): A Quality Improvement Program for Firearm Injury Prevention
Source: West J Emerg Med. 2020 Aug 20;21(5):1123–30. doi: 10.5811/westjem.2020.5.46952 (PMC7514406; doi:10.5811/westjem.2020.5.46952)
Supplement: Supplementary file 1 [file wjem-21-1123-s001.pdf]

Appendix Figure 1. Script for Providers for ED CALM intervention.

Advice to research coordinators: Deliver this intervention to all patients that rule in as long as they are not violent/threatening. Deliver the message as part of the care team (this is your role in this study)—you are providing valuable information for this patient cohort. If a patient tells you they do not want to listen to you, try to redirect them that you are here to help provide some brief, additional information for their safety and proceed with the intervention (again as long as the patients are not violent or verbally abusive). This can be a challenging patient cohort to work with. If you are unable to deliver the intervention, please be specific about the challenges on your data collection sheet so we can better troubleshoot in the future.

In ED, for patients who will be discharged:

Good morning/afternoon/evening, my name is _________. I am part of the ED team. Today, I would like to talk to you about dangerous items in your home, and what you and your family/friends can do once you are discharged to help keep you safe.

1) Do you currently have access to firearms or other potentially dangerous things in your home that you could use to hurt or kill yourself?

   (If yes) What are they? Where are they kept?

   (If no) Do you think you will have access to it?

2) Have you attempted suicide in the past? (alternative language: "have you tried to hurt yourself before?")

3) Is there a way for you to either lock the <u>(gun, pills, etc)</u> up safely or give them to a friend or family member for the time being?

   (If yes) What will you do or who will help you do this? Do you own a gun lock or gun safe?

   (If no) Would you be willing to store them somewhere else if you were given the option?

      (If yes- provide handout, show options from handout)

      (If no- provide handout explain the concern for their safety and the need to remove lethal means from their home/immediate surroundings.)

4) How long do you feel like you will be able to either lock these things up or give them to a friend or family member?

5) Do you have anyone in your life, like friends or family members, that you can talk to if your thoughts of suicide return?

6) Do you have a therapist or counselor that you can contact if your thoughts of suicide return?

   (Provide with handout of local resources, point out Lifeline phone number for 24 hr. access to crisis specialists)

As part of this plan to help keep you safe, someone from Washington University will call you in the next 2-3 days to check in and see how you are doing. We will also check to see if you were able to store your firearms outside of the home at that time

*If patient says no or is hesitant, explain that it will be a brief conversation just to check in on their well-being and to see if they were able to contact any personal or professional resources, or need more information.

7) Let's review what we just discussed: we will follow up with you over the phone on <u>DATE/TIME</u>. This phone conversation will come from a Washington University phone number, and will not be in any way connected to any bills or collections from Barnes Jewish Hospital. In the meantime, you're going to speak with <u>PERSON/AGENCY/RELATIONSHIP</u>, and remove any lethal means from your home by <u>METHOD/LOCATION</u> for <u>LENGTH OF TIME</u>.
   Patient contact information____
   ____
   In order to follow-up with your safety planning, we would like to also get the contact information for a friend, family member, or someone else who you trust that can confirm if you were able to successfully store your firearms outside of the home. Who would you like for us to contact?
   Family/friend contact information____
   ____

8) Do you have any further questions or concerns?

****If the patient endorses continued suicidality during your CALM intervention, please notify the patient's attending, as this could potentially change the patient's disposition

Time it took to complete intervention ____

Directions for Follow-Up phone call

Follow up via phone within 48-72 hours (up to 3 phone call attempts)

1. Did you store safely store things like firearms that could be potentially lethal in a suicide attempt inside or outside of the home?

   (If yes) Where/with whom were you able to do so? How long do you think that you will keep these things there?

   (If not) How come? What are the barriers keeping you from safely storing these things? Can we come up with a storage plan now?

2. Are you currently having thoughts of suicide?

   (If yes) Have you done anything to harm yourself/do you plan to harm yourself? (Go through suicide risk assessment below and determine if return to ER is necessary)

   (If no, proceed to next question)

3. Did you establish any follow up care?

   (If yes) Can you briefly explain what that is? Did you make an appointment with provider, like your primary care doctor, a psychiatrist or a therapist, or did you reach out to a friend/family member?

   (If no) What are the barriers keeping you from doing so? Can we come up with a safety plan now?

Suicide Risk Assessment

1) Are you having thoughts of suicide?
   a. If yes, encourage patient to return to the ED or outpatient psych office for re-evaluation and:
   b. Call Provident's suicidal crisis line that was created specifically for use in the CALM study at **314-647-5959**. Conference call into the Provident line and step off the line when the Provident counselor is on board. Please be prepared to give a "warm handoff" the following information to Provident in case the line is disconnected:
      i. Patient's name
      ii. Patient's phone number
      iii. Patient's physical location (if known)
      iv. Brief explanation of the situation (ex: "John was seen in the ED 2 days ago with thoughts of hurting himself, now continues to have these thoughts. We have called for additional help with managing his ongoing feelings of depression and hopelessness", etc).

2) Do you have a plan for suicide?
    a. If yes, encourage patient to return to the ED for re-evaluation and call Provident's suicidal crisis line that was created specifically for use in the CALM study at **314-647-5959**.
3) Do you have access to anything that you could use to hurt yourself?
    a. If yes, encourage safe storage of firearms, pills, etc, or encourage the patient to remove his/herself from situations where he/she is exposed to these dangerous items.
    b. Call Provident's suicidal crisis line that was created specifically for use in the CALM study at **314-647-5959**.
4) Have you done anything to hurt yourself since you left the emergency department?
    a. If yes, encourage the patient to return to the ED for re-evaluation
    b. Call Provident's suicidal crisis line that was created specifically for use in the CALM study at **314-647-5959**.
5) Can you stay safe while we're talking on the phone?
    a. If no, contact local PD/EMS to go to the home and evaluate the patient--call 911 immediately.
    b. If you are uncomfortable calling 911 on the patient, you can also call Provident's suicidal crisis line that was created specifically for use in the CALM study at **314-647-5959**.
