# Supplementary material for: Counseling on Access to Lethal Means-Emergency Department (CALM-ED): A Quality Improvement Program for Firearm Injury Prevention
Source: West J Emerg Med. 2020 Aug 20;21(5):1123–30. doi: 10.5811/westjem.2020.5.46952 (PMC7514406; doi:10.5811/westjem.2020.5.46952)
Supplement: Supplementary file 2 [file wjem-21-1123-s002.pdf]

Appendix Figure 2. When my friends or I need help, I will call…

Crisis/Distress Hotlines

Provident Life Crisis Services
314-647-4357

Behavioral Health Response
314-469-6644 (in St. Louis metro area)
1-800-811-4760

Kids Under Twenty One (KUTO)
314-644- 5886 (local)
1-888-644-5886

Safe Connections (Domestic Violence)
24-hour crisis line 314-531-2003

ALIVE (Alternatives to Living in Violent Environments)
314-993-2777

Trevor Helpline (LGBT youth)
866-488-7386

Youth Emergency Service Hotline
314-727-6294

National Suicide Prevention Lifeline
1-800-273-TALK (8255)

Hospitals for Psychiatric Needs

Barnes-Jewish Hospital
1 Barnes Jewish Hospital Plaza, St. Louis, MO 63110
314-747-3000

CenterPointe Psychiatric Hospital (open 24 hrs)
4801 Weldon Spring Pkway, Weldon Spring, MO 63304
800-345-5407

SSM St. Mary's Health Center
6420 Clayton Rd. Richmond Hts. MO 63117
314-768-8000

DePaul Health Center
12303 De Paul Dr, Bridgeton, MO 63044
314-344-6000

Counseling Services

BJC Behavioral Health
1430 Olive, Suite 500
St. Louis, MO 63103-2377
314-206-3700

Provident, Inc.
2650 Olive Blvd. St Louis, MO 63103
314-533-8200

Counseling Services (cont.)

Hopewell
1504 S. Grand Blvd. St Louis, MO 63104
314-531-1770

Jewish Family and Children's Service
10950 Schuetz Rd, St. Louis, MO 63146
314-993-1000

Lutheran Family and Children's Services of Missouri
9666 Olive Boulevard Suite 400 St. Louis, MO 63132
314-787-5100

St. Louis Center for Family Development
4236 Lindell Blvd # 200, St. Louis, MO 63108
314-531-1155

Drug and Alcohol Abuse

NCADA (National Council on Alcoholism and Drug Abuse)
314-962-3456 (County); 314-664-7550 (City)

New Beginnings C-Star
314-367-8989

Bridgeway Behavioral Health
314-226-9030

Preferred Family
314-772-2205 (City)
314-729-7050 (South Co.); 314-972-8132 (North Co.)

Queen of Peace (women only)
314-531-0511

St. Patrick Center
314-802-0693

General Information/Emergency Services

United Way (referrals to resources and services)
2-1-1 or 314-424-INFO (4636)

Police/Emergency Assistance
9-1-1

St. Louis City Police non-emergency number
314-231-1212

St. Louis County Police non-emergency number
636-529-8210

Child Abuse/Neglect Line
1-800-392-3738

Washington University in St. Louis
Institute for Public Health
